# Case Report: Let Us Not Forget the Treatment That Some Patients Have Received—The Brief 50-Year History of a Kidney Transplant Survivor

**DOI:** 10.3389/fmed.2022.906925

**Published:** 2022-05-24

**Authors:** Espen Nordheim, Melinda Raki, Karsten Midtvedt

**Affiliations:** ^1^Department of Transplantation Medicine, Section of Nephrology, Oslo University Hospital, Rikshospitalet, Oslo, Norway; ^2^Department of Nephrology, Oslo University Hospital, Oslo, Norway; ^3^Department of Pathology, Oslo University Hospital, Rikshospitalet, Oslo, Norway

**Keywords:** kidney, transplantation - kidney, biopsy, immunosuppressants, history

## Abstract

**Background:**

There has been a considerable improvement in post-transplant care since the early 1960s. Some patients we meet in the clinic have personally experienced this progress and have histories to tell that one must not forget. This is the brief history of a long-time “transplant survivor.”

**Case Presentation:**

In 1970, a young woman developed acute oedema, proteinuria, hypertension and oliguria during pregnancy. Labor was induced, but neither the child nor the kidney function could be saved. Our patient started dialysis, and 4 years later received a kidney transplant donated by her father (then 55 years of age). Maintenance immunosuppression consisted of prednisolone and azathioprine until 2011, when azathioprine was switched to everolimus due to skin cancer. Before this, our patient was highly satisfied with prednisolone/azathioprine, despite discussions regarding newer immunosuppressive drugs, and always reminded the treating physician that one should “never change a winning team.” Retrospectively, the avoidance of calcineurin inhibitors might have been beneficial for this patient who still has preserved an excellent renal function with s-creatinine levels around 100 μmol/L and just had sparse fibrosis detected in a recently performed transplant biopsy. The transplanted kidney is now 101 years old and is still working 24/7.

**Conclusions:**

Our patient received a kidney transplant for 46 years ago and still has a remarkably stable transplant function with s-creatinine levels around 100 μmol/L. This case report illustrates the potential endurance of the kidneys and is a reminder to keep taking individualized treatment decisions even though new treatment alternatives promise superiority.

## Background

**Figure 1:**
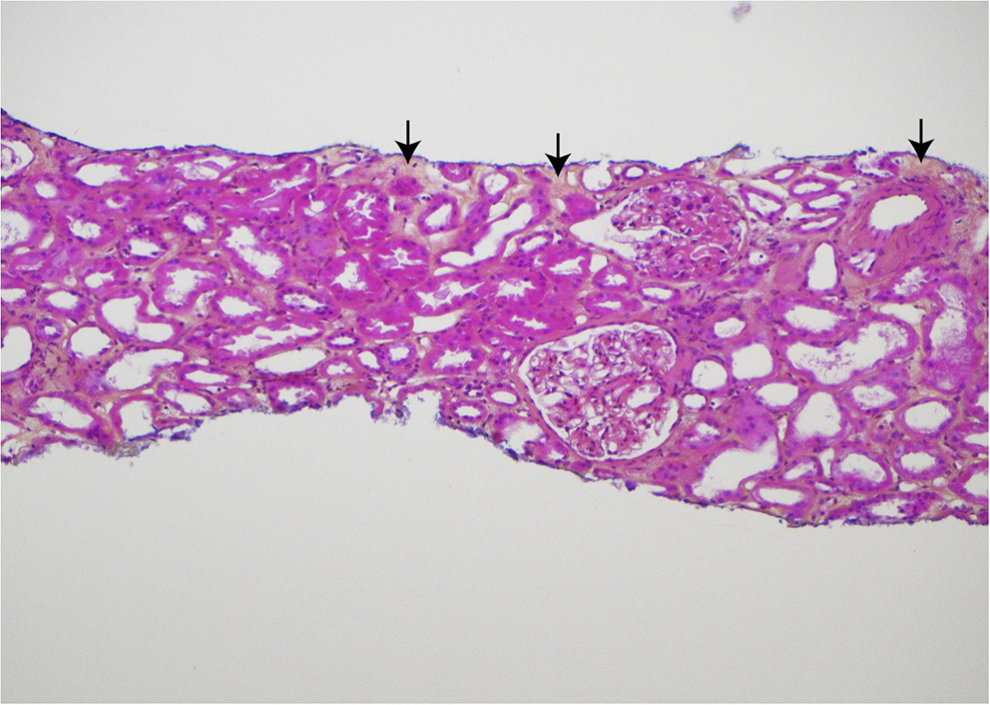
Histologic findings in the core needle biopsy of the 101-year old kidney transplant, sampled April 2020. Hematoxylin, eosin, and saffron (HES) stained section demonstrating only sparse, focal interstitial fibrosis (yellow areas with arrows). There is no interstitial inflammation and only a slight, segmental increase of the mesangial matrix in some glomeruli. Original magnification ×100. Published in agreement with the patient.

Recently, a 72-year-old Caucasian woman who has been followed at our unit for 50 years came for a regular out- patient visit. She developed renal failure in 1970 and received a kidney transplant in 1974. Her kidney transplant has been well- functioning ever since, despite 46 years' treatment with immunosuppressive medication. **In April 2020 when the kidney transplant had passed 101 years of age, a biopsy was taken (****Figure 1****)**, demonstrating only sparse fibrosis.

This is the brief history of a long-time transplant survivor.

## Case Presentation

**Our patients' medical history started in 1970** when she was pregnant (para 1). At the end of the last trimester, she developed oedema and proteinuria without signs of hypertension earlier during the pregnancy. Six days before the estimated time of delivery, she developed severe vaginal bleedings, hypertension (150/130 mmHg), proteinuria (2 g/24 h), oedema and eventually oliguria. Placental bleeding was suspected leading to an emergency induced labor, which resulted in stillbirth. Post-delivery blood pressure stabilized without antihypertensive treatment, but oliguria persisted and eventually our patient became anuric, thus peritoneal dialysis was started.

**Figure 2:**
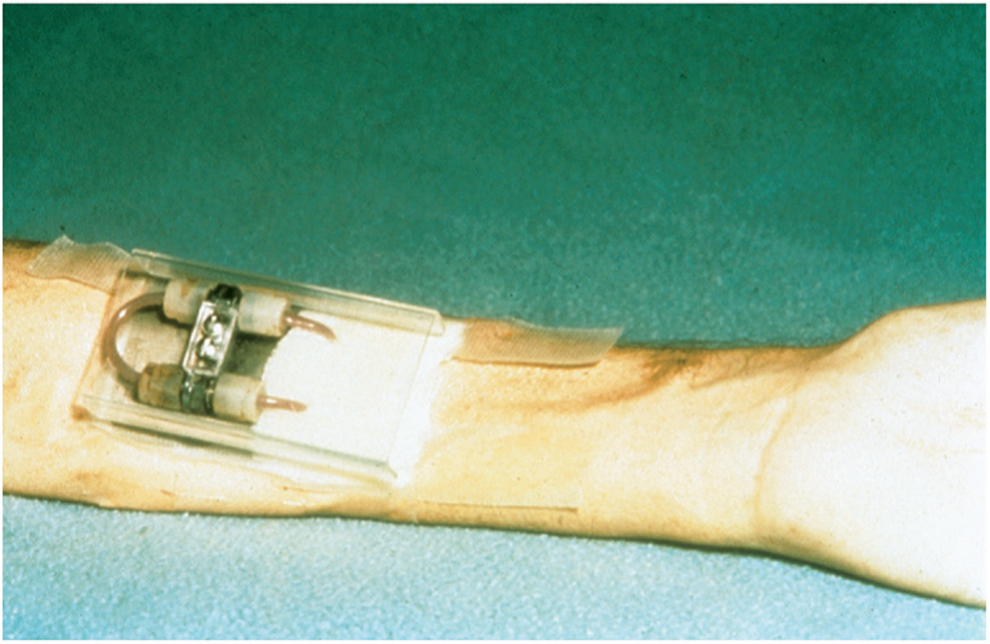
“Schribner shunt” in place, at the left arm of the patient after 4 weeks attached to a stainless steel arm plate protected by a plastic cover placed over the shunt. Reproduced from Quinton et al. (1) with permission of Wolters Kluwer Health, Inc.

As the clinical presentation was considered atypical for pregnancy- related kidney disease, it was decided to perform a kidney biopsy. After the first attempt with a blindly sampled percutaneous procedure not obtaining any representative material, an open biopsy procedure was chosen for the second attempt. The pathologists described generalized cortical necrosis in the kidney biopsies, thought to be caused by severe pre- eclampsia. Urine production gradually increased and dialysis could be halted after about 5 weeks. After cessation of dialysis, renal function was stable with creatinine clearance levels around 15–16 ml/min and proteinuria 1.1 g/24 h. Blood pressure levels remained elevated at 160–180/100–110 mmHg, but no antihypertensive treatment was started. At a routine consultation in October 1973, the treating physician described her as “*wellbeing*” even though hemoglobin level of 4.7 g/dl and s-creatinine at 1122 μmol/L (12.7 mg/dl) was remarked. Our patient was informed to start oral iron supplementation and that ….*there was an indication for kidney transplantation!* Subsequently, pre- transplant work- up was initiated what included evaluation of family members as potential donors. The father of our patient (then aged 55) was accepted as donor and the transplantation was scheduled for January 1974. Human Leucocyte Antigen (HLA) - typing for HLA-A and HLA-B was performed in both donor and recipient and two HLA-mismatches were found, which was categorized as a D-match. Our patient needed to restart dialysis 2 months before the scheduled transplantation; at this point haemodialysis *via* an arterial- venous shunt (1) (Figure 2) was chosen.

**The kidney transplantation performed January 1974** included simultaneous bilateral nephrectomy common at the time (2). An accidental bleeding during the transplant procedure led to a per-operative splenectomy. Total cold ischemia time of 44 min was registered for the kidney transplant and 3,000 ml infusion fluids were given to the transplant recipient together with 300 mg hydrocortisone and 175 mg azathioprine as initial immunosuppression.

A clinical rejection was suspected on post- transplant day 6 due to an increase in s-creatinine- from 106 μmol/L (1.2 mg/dl) to 150 μmol/L (1.7 mg/dl). Anti- rejection treatment consisting of 5 gram intravenous methylprednisolone and radiation therapy [150 Roentgen × 3 (equivalent to 1.5 Gy × 3)] was started without histological verification of the rejection diagnosis. Renal function stabilized [creatinine 115 μmol/L (1.3 mg/dl)] after the rejection episode and the patient was discharged at day 12 with the following daily medication: prednisolone 50 mg q.d, azathioprine 175 m g q.d., furosemide 40 mg t.d.s. and no anti-hypertensive treatment.

**At the clinical visit at 1 year after transplantation** she reported to be very well. The clinician noted cushingoid characteristics, 124/60 mmHg blood pressure and creatinine clearance 93 ml/min. Our patient was informed to continue following medication: prednisolone 175 mg q.d., azathioprine 225 mg q.d and furosemide 40 mg q.d, in addition to iron supplements and antacids. Eighteen months after transplantation the prednisolone dose was tapered to 10 mg q.d. and azathioprine dose to 100 mg q.d.

**The following years** went without any specific concerns. Renal function remained stable with serum creatinine values around 105 μmol/L (1.3 mg/dl).

From 15 years on after transplantation a broad specter of skin manifestations was diagnosed and treated: solar keratosis, fibroepithelial polyps, seborrheic keratosis, nodular basal cell carcinoma and squamous cell carcinoma. The different skin lesions slowly improved after azathioprine was switched to everolimus, an inhibitor of the mammalian target of rapamycin (mTORi) in 2011 (trough 4–8 μg). After the drug switch, serum cholesterol levels increased, followed by intensified lipid- lowering therapy. In 2017, she developed symptoms of angina pectoris. Coronary angiography revealed left coronary artery stenosis and a drug eluting stent was successfully implanted. Bone density has been measured regularly. The first signs of osteopenia were registered in 1998 and regional osteoporosis was diagnosed in 2019.

In April 2020, her blood pressure was 124/60 mmHg and serum creatinine value was 113 μmol/L. Current medication consisted of prednisolone 5 mg × 1, Everolimus 1 mg × 2, acetylsalicylic acid 75 mg × 1, rosovastatin 10 mg, ezetimib 10 mg, in addition to a combination of calcium and vitamin D at 1,000 mg/800 units.

## Discussion

This patient is a living witness of modern nephrology history. Hemoglobin levels below 5 g/dl due to renal anemia was treated with blood-transfusions and iron supplements in the 1960–70s; recombinant erythropoietin arrived on the marked in the late 1980s (3). Blood access for receiving haemodialysis prior to transplantation was achieved through an indwelling shunt placed externally on the forehand (Figure 2). The first kidney transplantation in Norway was performed in 1956, but the official transplant program was only 6 years old when our patient was transplanted in 1974.

Short and long-term outcome following kidney transplantation in the 70s was poor. One-year rejection rates were 70%−80% while the 1- and 5-year patient survival was 60 and 45%, respectively (4). In 1974, the pre- transplant immunological testing was restricted to HLA-A and HLA-B phenotyping in addition to cross- matching, and mismatches were graded from A to G in most Scandinavian centers (5). Four years later, after the introduction of HLA-DR typing; 1-year graft survival was 55% for HLA-DR incompatible kidney transplants and 87% for HLA-DR compatible transplants in our center (5). Prednisolone and azathioprine were the only two immunosuppressive drugs available in transplantation at the time. One dose fitted all and individual azathioprine treatment, based on 6-thioguanine nucleotides (6-TGN) - monitoring, were still two decades away (6). Radiation therapy and 5 g of methylprednisolone was used for treatment of rejection suspected from clinical markers alone; more standardized rejection criteria based on histology findings was not introduced until 1993 (7). Radiation treatment has later been abandoned in kidney transplantation (8). Even though methylprednisolone is still in use, the recommended doses are much lower and usually only utilized in the case of biopsy-proven rejection.

In this early transplant era, 15% of the patients died of infections during the first year in our center. Pneumocystis jiroveci prophylaxis was not routinely applied in kidney transplant recipients until late 1990's.

**Figure 3:**
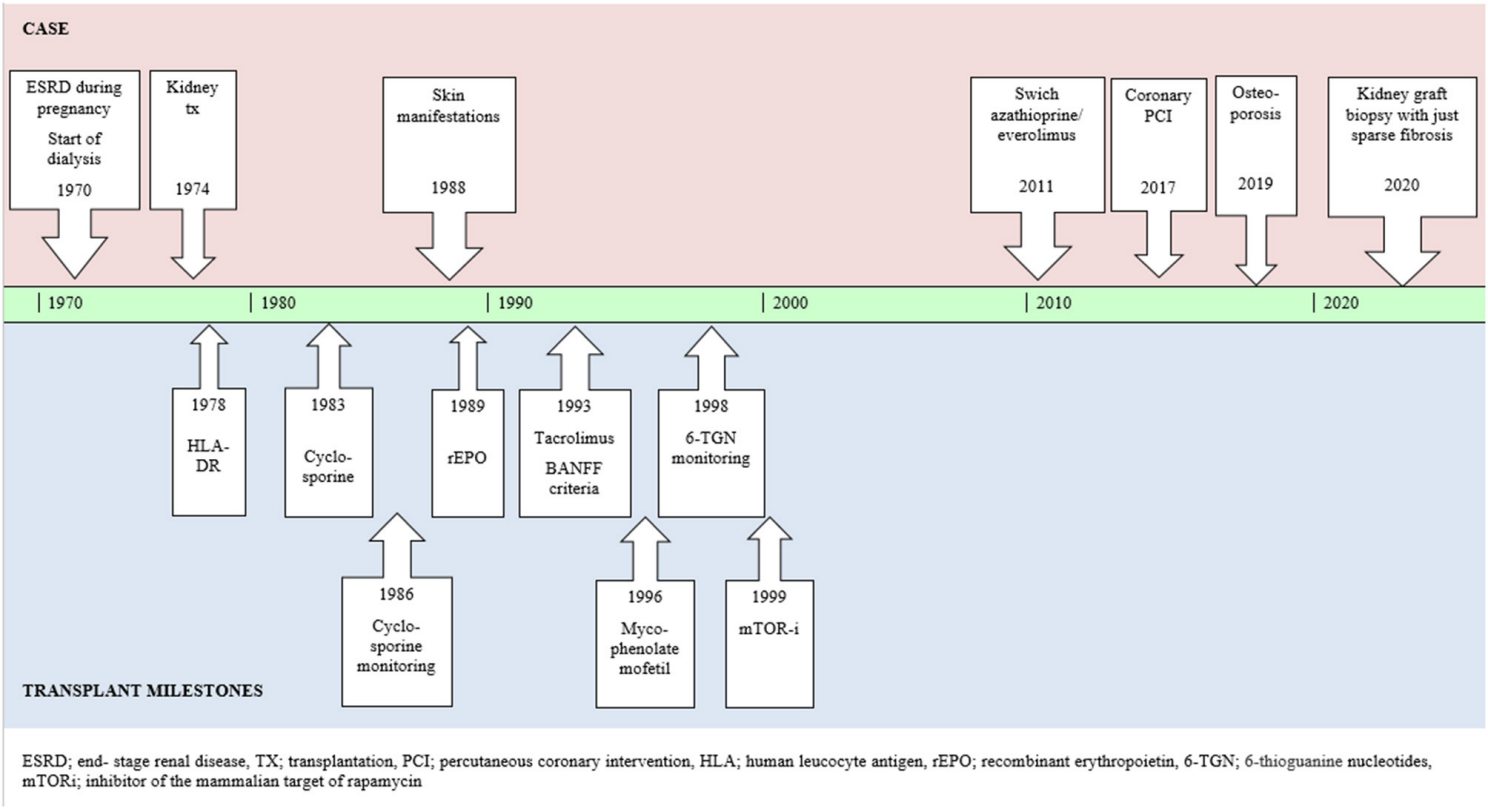
Case report summarized in timeline including relevant milestones of the kidney transplant history.

Switches to “new and better” immunosuppressive treatment was repeatedly discussed with the patient as cyclosporine (1983), tacrolimus (1993) and mycophenolate mofetil (1996) became available (Figure 3). However, our patient felt confident with her treatment and did not want to “take the risk” of changing a medication she experienced as safe and was familiar with. Retrospectively, avoidence of the nephrotoxic calcineurin-inhibitors might have been beneficial for our patient to preserve excellent renal function.

The introduction of the calsinurin inhibitors (CNI) cyclosporine/tacrolimus was of significant importance improved graft and patient survival following kidney transplantation (9–12). Shortly after the introduction of cyclosporin Myers et al. (13) demonstated how “long- term” use of cyclosporin was associated with an irreversible deterioration of renal function due to tubulo- intestinal injury and glomerulosclerosis. These findings have been confirmed by others both for cyclosporin and tacrolimus (14–17). One must remember that in this early phase of CNI use the dosing was much higher and often in mg/kg and not according to measured concentration (trough values) The concept of CNI- toxicity is multifactorial with both demografic and pharmacogenetic flexibilty and is still beeing discussed (18).

Calcineurin inhibitors are still the cornerstones in maintenance immunosuppression after kidney transplantation; and tacrolimus has largely become the first choice due to better tolerability, rejection prevention and graft survival. Low- dose tacrolimus protocols have been implemented in several centers after it was found safe and advantageous for renal function when combined with mycophenolate mofetil and corticosteroids after renal transplantation (19, 20). A tacrolimus- based immunosuppressive regime was given to over 90% of new adult kidney transplant recipients in the United States in 2020 (21). CNI- free protocols after renal transplantation are available, which includes mTOR- inhibitors (22) or belatacept (23) but often lead to more rejections.

Our patient did, however, switch from azathioprine to everolimus in 2011 after being treated for severeal skin cancers, as the mTORs then had demonstrated a possible reduced risk for skin cancer (24).

After this swich, a severe worsening of her blood lipid profile was registered, a well-known side- effect of everolimus (25). Fluvastatin was initiated in order to reduce the cardiovascular risk (26) and later replaced with rosuvastatin (27). Despite these preventive efforts, our patient developed symptomatic angina, which was efficiently treated with percutaneous coronary intervention in 2017.

This kidney transplant has been through 101 rough years, but still there is only sparse fibrosis in the recent transplant biopsy which by our pathology unit was evaluated as a normal kidney transplant biopsy according to the Banff classification: The s-creatinine remains at levels around 100 μmol/L (1.1 g/dl) and just sparse proteinuria has been registered. It is out of the range for this report to answer how old a transplanted kidney can get, but we do think this case illustrates which endurance the kidneys might have but also that the expression “never change a winning team” might be relevant in the navigation of different immunosuppressive regimens in the follow-up of kidney transplant recipients.

The story doesn't end here but goes on and just like in the fairytales … *the patient and her transplanted kidney lived happily ever after……*

## Data Availability Statement

The original contributions presented in the study are included in the article/supplementary material, further inquiries can be directed to the corresponding author.

## Ethics Statement

Written informed consent was obtained from the individual(s) for the publication of any potentially identifiable images or data included in this article.

## Author Contributions

EN, MR, and KM created the idea and reviewed and finished the manuscript. EN drafted the manuscript. All authors have read and approved the manuscript.

## Conflict of Interest

The authors declare that the research was conducted in the absence of any commercial or financial relationships that could be construed as a potential conflict of interest.

## Publisher's Note

All claims expressed in this article are solely those of the authors and do not necessarily represent those of their affiliated organizations, or those of the publisher, the editors and the reviewers. Any product that may be evaluated in this article, or claim that may be made by its manufacturer, is not guaranteed or endorsed by the publisher.

## Abbreviations

HLA: human leukocyte antigen
mTOR: inhibitor of the mammalian target of rapamycin
6-TGN: 6-thioguanine nucleotides.

## References

[1] Quinton W Dillard D Scribner BH. Cannulation of blood vessels for prolonged hemodialysis. Trans Am Soc Artif Intern Organs. (1960) 6:104–13. 13738750

[2] Midtvedt K Hartmann A Bentdal O Brekke IB Fauchald P. Bilateral nephrectomy simultaneously with renal allografting does not alleviate hypertension 3 months following living-donor transplantation. Nephrol Dial Transplant. (1996) 11:2045–9. 10.1093/oxfordjournals.ndt.a027094 8918720

[3] Eschbach JW Abdulhadi MH Browne JK Delano BG Downing MR Egrie JC. Recombinant human erythropoietin in anemic patients with end-stage renal disease. Results of a phase III multicenter clinical trial. Ann Intern Med. (1989) 111:992–1000. 10.7326/0003-4819-111-12-992 2688507

[4] Albrechtsen D Pfeffer PF. En Gave For Livet. Vol 1. Bergen: Vigmostad & Bjørke AS (2011), p. 58–60.

[5] Albrechtsen D Flatmark A Jervell J Solheim B Thorsby E. HLA-DR antigen matching in cadaver renal transplantation. Lancet. (1978) 1:825. 10.1016/S0140-6736(78)93026-X 85842

[6] Bergan S Rugstad HE Bentdal O Sødal G Hartmann A Leivestad T. Monitored high-dose azathioprine treatment reduces acute rejection episodes after renal transplantation. Transplantation. (1998) 66:334–9. 10.1097/00007890-199808150-00010 9721802

[7] Solez K Axelsen RA Benediktsson H Burdick JF Cohen AH Colvin RB. International standardization of criteria for the histologic diagnosis of renal allograft rejection: the Banff working classification of kidney transplant pathology. Kidney Int. (1993) 44:411–22. 10.1038/ki.1993.259 8377384

[8] Godfrey AM Salaman JR. Radiotherapy in treatment of acute rejection of human renal allografts. Lancet. (1976) 1:938–9. 10.1016/S0140-6736(76)92715-X 57340

[9] Calne RY White DJ Thiru S Evans DB McMaster P Dunn DC. Cyclosporin A in patients receiving renal allografts from cadaver donors. Lancet. (1978) 2:1323–7. 10.1016/S0140-6736(78)91970-0 82836

[10] Calne RY Rolles K White DJ Thiru S Evans DB McMaster P. Cyclosporin A initially as the only immunosuppressant in 34 recipients of cadaveric organs: 32 kidneys, 2 pancreases, and 2 livers. Lancet. (1979) 2:1033–6. 10.1016/S0140-6736(79)92440-1 91781

[11] Starzl TE Todo S Fung J Demetris AJ Venkataramman R Jain A. 506 for liver, kidney, and pancreas transplantation. Lancet. (1989) 2:1000–4. 10.1016/S0140-6736(89)91014-3 2478846 PMC2966318

[12] Fung JJ Abu-Elmagd K Todo S Shapiro R Tzakis A Jordan M. Overview of FK506 in transplantation. Clin Transpl. (1990) 115–21. 1715740 PMC3079459

[13] Myers BD Ross J Newton L Luetscher J Perlroth M. Cyclosporine-associated chronic nephropathy. N Engl J Med. (1984) 311:699–705. 10.1056/NEJM198409133111103 6382005

[14] Palestine AG Austin HA 3rd Balow JE Antonovych TT Sabnis SG Preuss HG. Renal histopathologic alterations in patients treated with cyclosporine for uveitis. N Engl J Med. (1986) 314:1293–8. 10.1056/NEJM198605153142005 3702930

[15] Klintmalm G Bohman SO Sundelin B Wilczek H. Interstitial fibrosis in renal allografts after 12 to 46 months of cyclosporin treatment: beneficial effect of low doses in early post-transplantation period. Lancet. (1984) 2:950–4. 10.1016/S0140-6736(84)91166-8 6149343

[16] Starzl TE Fung J Jordan M Shapiro R Tzakis A McCauley J. Kidney transplantation under FK 506. JAMA. (1990) 264:63–7. 10.1001/jama.264.1.63 1693970 PMC2979318

[17] Randhawa PS Shapiro R Jordan ML Starzl TE Demetris AJ. The histopathological changes associated with allograft rejection and drug toxicity in renal transplant recipients maintained on FK506. Clinical significance and comparison with cyclosporine. Am J Surg Pathol. (1993) 17:60–8. 10.1097/00000478-199301000-00007 7680544 PMC3229279

[18] Xia T Zhu S Wen Y Gao S Li M Tao X. Risk factors for calcineurin inhibitor nephrotoxicity after renal transplantation: a systematic review and meta-analysis. Drug Des Devel Ther. (2018) 12:417–28. 10.2147/DDDT.S149340 29535503 PMC5836651

[19] Ekberg H Tedesco-Silva H Demirbas A Vítko S Nashan B Gürkan A. Reduced exposure to calcineurin inhibitors in renal transplantation. N Engl J Med. (2007) 357:2562–75. 10.1056/NEJMoa067411 18094377

[20] Størset E Åsberg A Hartmann A Reisaeter AV Holdaas H Skauby M. Low-target tacrolimus in de novo standard risk renal transplant recipients: a single-centre experience. Nephrology. (2016) 21:821–7. 10.1111/nep.12738 26854648

[21] Lentine KL Smith JM Hart A Miller J Skeans MA Larkin L. OPTN/SRTR 2020 annual data report: kidney. Am J Transplant. (2022) 22(Suppl 2):21–136. 10.1111/ajt.16982 35266618

[22] Holdaas H Rostaing L Serón D Cole E Chapman J Fellstrøm B. Conversion of long-term kidney transplant recipients from calcineurin inhibitor therapy to everolimus: a randomized, multicenter, 24-month study. Transplantation. (2011) 92:410–8. 10.1097/TP.0b013e318224c12d 21697773

[23] Vincenti F Charpentier B Vanrenterghem Y Rostaing L Bresnahan B Darji P. A phase III study of belatacept-based immunosuppression regimens versus cyclosporine in renal transplant recipients (BENEFIT study). Am J Transplant. 2010;10:535–4611 10.1111/j.1600-6143.2009.03005.x 20415897

[24] Campistol JM Eris J Oberbauer R Friend P Hutchison B Morales JM. Sirolimus therapy after early cyclosporine withdrawal reduces the risk for cancer in adult renal transplantation. J Am Soc Nephrol. (2006) 17:581–9. 10.1681/ASN.2005090993 16434506

[25] Budde K Becker T Arns W Sommerer C Reinke P Eisenberger U. Everolimus-based, calcineurin-inhibitor-free regimen in recipients of de-novo kidney transplants: an open-label, randomised, controlled trial. Lancet. (2011) 377:837–47. 10.1016/S0140-6736(10)62318-5 21334736

[26] Holdaas H Fellström B Jardine AG Holme I Nyberg G Fauchald P. Effect of fluvastatin on cardiac outcomes in renal transplant recipients: a multicentre, randomised, placebo-controlled trial. Lancet. (2003) 361:2024–31. 10.1016/S0140-6736(03)13638-0 12814712

[27] Robertsen I Asberg A Granseth T Vethe NT Akhlaghi F Ghareeb M. More potent lipid-lowering effect by rosuvastatin compared with fluvastatin in everolimus-treated renal transplant recipients. Transplantation. (2014) 97:1266–71. 10.1097/01.TP.0000443225.66960.7e 24521776 PMC4127422

